# Thermal Deformation Measurement of the Surface Shape of a Satellite Antenna Using High-Accuracy Close-Range Photogrammetry

**DOI:** 10.3390/s24144722

**Published:** 2024-07-20

**Authors:** Kaifeng Ma, Guiping Huang, Junzhen Meng

**Affiliations:** College of Surveying and Geo-Informatics, North China University of Water Resources and Electric Power, Zhengzhou 450046, China; huangguiping123@163.com (G.H.); mengjunzhen@ncwu.edu.cn (J.M.)

**Keywords:** close-range photogrammetry, thermal deformation measurement, satellite antenna, high accuracy

## Abstract

To determine both the size of a satellite antenna and the thermal deformation of its surface shape, a novel high-accuracy close-range photogrammetric technique is used in this study. The method is also applied to assess the performance of the antenna in orbit. The measurement principle and solution method of close-range photogrammetry were thoroughly investigated, and a detailed measurement test scheme was developed. A thermal deformation measurement of the surface shape of a satellite antenna was then carried out. The results show that the measurement error using close-range photogrammetry was smaller than 0.04 mm, which meets the accuracy requirement. Thanks to the high accuracy, it was discovered that both the surface shape and the rib precision of the satellite antenna deteriorate with decreasing temperature. The accuracy of the surface shape and ribs was lowest when the temperature node was −60 °C. The maximum root mean square errors (RMSEs) reached 0.878 mm and 0.761 mm, respectively. This indicates that the surface shape deformation error of the antenna caused by high and low temperatures is relatively high. However, the requirement for the technical design index (RMSE ≤ 1 mm for the surface shape accuracy of the antenna) is still met. Furthermore, for temperature differences of 40 °C and 80 °C, the measured RMSEs for the surface shape deformation were 0.216 mm and 0.411 mm, respectively. Overall, the technical design indicators (RMSE ≤ 0.3 mm and RMSE ≤ 0.5 mm, respectively) for the surface shape deformation of the antennas are met.

## 1. Introduction

In the course of satellite operation in orbit, it will be particularly affected by various special factors such as the alternating hot and cold of the space environment, which will lead to some deformation of the antenna shape in this vacuum environment of alternating hot and cold. The surface shape deformation of satellite antennas due to large temperature variations is one of the most important technical indicators. Its value directly reflects both the quality and the operational performance of the satellite components. Therefore, before a satellite is launched into orbit, it must be tested on the ground in a simulated space environment using standard thermal deformation tests to ensure good design and manufacturing quality [1]. Accurate testing ensures that a satellite antenna will perform as expected and makes it easier to identify improvements.

Considering the large temperature difference combined with the requirement for high accuracy of a surface shape measurement, it is necessary to use a very accurate measurement technique to determine the surface shape. Conventional measurement methods such as the template method, lathe method, theodolite steel tape rule method, and double pentaprism method face many challenges under these conditions. These methods are often limited by the antenna orientation, measurement speed/accuracy/efficiency, and measurement range. In addition, measurement automation is difficult to achieve [2]. Overall, the extreme requirements for the efficient and accurate measurements of modern flexible satellite antennas are difficult to meet and new methods are urgently needed [3].

According to the literature [4,5,6], there are mainly eight kinds of modern large-scale coordinate measurement methods: the coordinate measuring machine (CMM), theodolite surveying system, total station surveying system, laser-tracking measurement system, laser-scanning measurement system, articulated arm-measuring machine, indoor GPS (iGPS), and close-range photogrammetry. These methods have their own characteristics and advantages in terms of method, accuracy, range, and portability. They are widely used in different areas, environments, and for a wide range of objects. Among them, close-range photogrammetry can better adapt to extreme environmental conditions and perform real-time dynamic measurements. It has been used in aerospace, heavy-industry manufacturing, and other commercial areas [7,8,9,10,11,12]. It can effectively perform the high-accuracy location, assembly, detection, and measurement of large-scale industrial components. According to the inspection requirements and conditions at various stages of the space engineering project, combined with the characteristics of the target object of the studied ribbed unfolded mesh satellite antenna and the peculiarity of the high and low temperatures, this non-contact, fast, efficient, and advanced new type of close-range photogrammetry technology can show its advantages and effectively meet the measurement requirements of this special task.

In recent years, several successful thermal vacuum deformation measurements of satellite antennas, using close-range photogrammetry, have been performed by different research groups [13,14,15,16,17]. Examples include the European Space Agency (ESA/ESTEC), Japan’s JAXA, the National Aeronautics and Space Administration (NASA), the United States Lockheed Martin Space Systems, Germany’s IABG, Alcatel Aerospace Company, Boeing Satellite Systems, and Northrop Grumman. They have performed thermal deformation measurements on satellite antennas and space telescopes. Their measurement technologies represent the highest level in spacecraft deformation measurement [14,15,18,19,20,21,22]. Based on studies reported in recent years, researchers in China have succeeded in measuring the thermal vacuum deformation of satellite antennas [23,24,25]. However, most of these studies on close-range photogrammetric techniques have been performed under thermal vacuum environmental conditions. Additionally, the antenna surface shape deformation is measured by fixing the guarded camera inside the thermal vacuum chamber, or placing the camera at an observation window outside the thermal vacuum chamber. Compared to the previous thermal vacuum environment in a more confined space, the protection conditions of the camera and the geometry of the photographic space in the conventional high–low temperature environment have been optimized, with certain advantages for optimizing and improving the accuracy of the photogrammetric results. However, there are few reports on the study of close-range photogrammetry techniques for satellite antenna surface shape deformation measurement using a single high-accuracy measuring camera in a high–low temperature environment with conventional air pressure. Aiming at the actual conditions and measurement requirements of a rib-spread mesh satellite antenna with a diameter of about 4 m, a measurement experiment of the deformation of an antenna surface shape in a high–low temperature environment by using close-range photogrammetry technology was proposed. The applicability and high accuracy of the close-range photogrammetry technology were verified, and the deformation size and regularity of the satellite antenna surface shape in a high–low temperature environment were obtained.

## 2. Principles of Close-Range Photogrammetry

**Figure 1:**
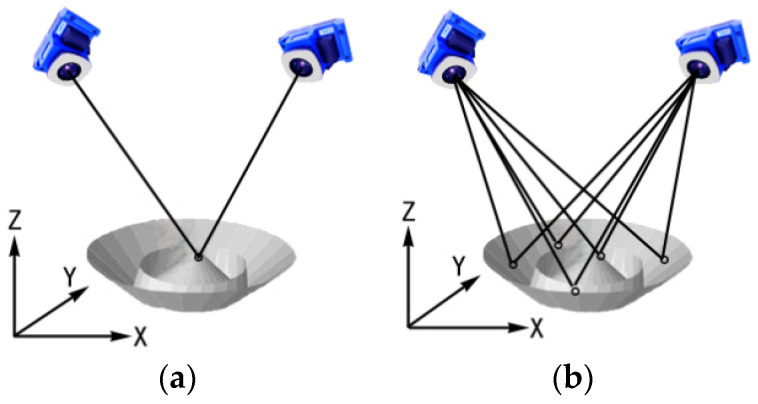
Fundamental principle of photogrammetry. (**a**) Single point triangulation; (**b**) multipoint triangulation.

Close-range photogrammetry is based on optical image information of the measured object obtained from camera photography. The three-dimensional spatial coordinates of the measured object are obtained by a measurement adjustment calculation. When the measured object is photographed, at least two or more high-resolution cameras should take pictures of the measured object simultaneously, or one camera should take pictures at different positions; see Figure 1. This produces two or more two-dimensional images of the target. The images are then subjected to digital processing such as image matching, triangulation and adjustment to produce accurate three-dimensional spatial coordinates of the object. The two-dimensional images produce two-dimensional coordinates in the image plane coordinate system. In a photogrammetric coordinate system, the two-dimensional coordinates are converted into two observation angles of the object using the photogrammetric principal distance parameter. The measurement principle is therefore the same as a theodolite measurement system [26], both of which use triangulation (Figure 1) to measure the three-dimensional coordinates of the target points. However, as photographic cameras cannot achieve the precise mutual alignment of theodolites, bundle adjustment is typically used to determine the precise orientation between the cameras. As there is some redundancy in the simultaneous measurement of multiple target points by cameras at different station positions, the position and attitude of the targets and between the cameras can also be obtained simultaneously.

**Figure 2:**
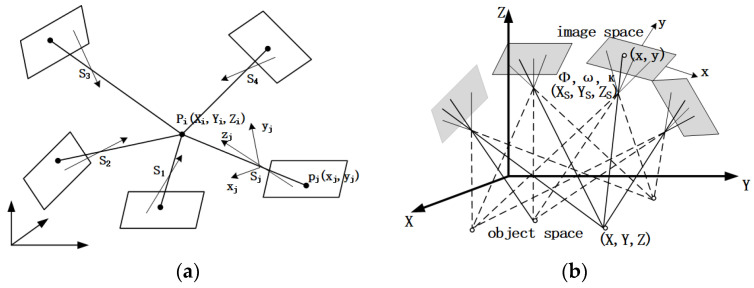
Multi-station photogrammetry and intersection. (**a**) The intersection of j camera stations; (**b**) Multi-station photogrammetry.

When two or more cameras are used to capture the target at different camera stations, the multi-view stereo model is formed from the intersection of multiple camera stations. As shown in Figure 2, the object point $P_{i}$ is formed by the intersection of j camera stations or j rays, and the j collinear equations (Equation (1)) can be formulated using the collinear condition.

(1) $$\{\begin{array}{c} x_{ij}-x_{0_{j}}+\Delta x_{ij}=-f_{j}\frac{a_{1_{j}}\left(X_{i}-X_{S_{j}}\right)+b_{1_{j}}\left(Y_{i}-Y_{S_{j}}\right)+c_{1_{j}}\left(Z_{i}-Z_{S_{j}}\right)}{a_{3_{j}}\left(X_{i}-X_{S_{j}}\right)+b_{3_{j}}\left(Y_{i}-Y_{S_{j}}\right)+c_{3_{j}}\left(Z_{i}-Z_{S_{j}}\right)}\\ y_{ij}-y_{0_{j}}+\Delta y_{ij}=-f_{j}\frac{a_{2_{j}}\left(X_{i}-X_{S_{j}}\right)+b_{2_{j}}\left(Y_{i}-Y_{S_{j}}\right)+c_{2_{j}}\left(Z_{i}-Z_{S_{j}}\right)}{a_{3_{j}}\left(X_{i}-X_{S_{j}}\right)+b_{3_{j}}\left(Y_{i}-Y_{S_{j}}\right)+c_{3_{j}}\left(Z_{i}-Z_{S_{j}}\right)} \end{array}$$

where $x_{0}$, $y_{0}$, and f are the inner orientation elements of the image, representing the principal points and the principal distance of the image, respectively. These parameters determine the position of the photographic center relative to the image and are used to restore the shape of the photographic beam during photography. $X_{s}$, $Y_{s}$, $Z_{s}$, ϕ, ω, and κ are the outer orientation elements of the image, which are parameters used to express the spatial position and attitude of the image or the photographic beam at the moment of photography. Furthermore, $a_{i}$, $b_{i}$, and $c_{i}$ (i=1,  2,  3) are the nine directional cosines, composed of the three external orientation elements ϕ, ω, and κ of the image, while Δx,  Δy are small deviations (distortions) of the coordinates of the respective image points with respect to their theoretical position coordinates (*x*, *y*).

The error equation is given by Equation (1). The principle of bundle adjustment is used to solve the solution, and the three-dimensional space coordinates ($X_{i},\;Y_{i},\;Z_{i}$) of the object points can be obtained [4].

## 3. Implementation

### 3.1. Test Object

**Figure 3:**
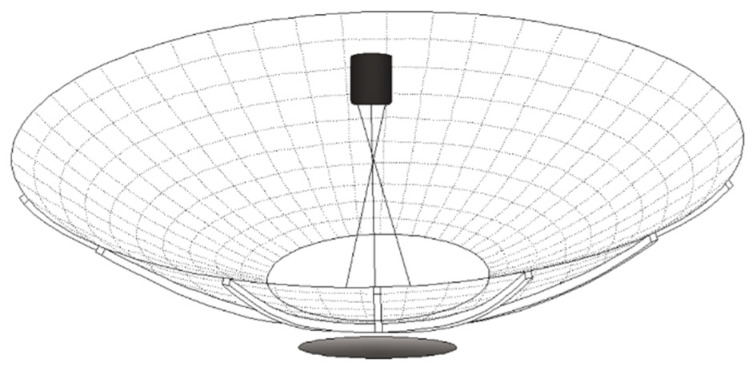
Deployable antenna with radial ribs.

The test object is a ribbed deployable mesh satellite antenna of approximately 4 m diameter. Its surface shape is similar to that shown in Figure 3.

It is a kind of umbrella-shaped antenna, and its reflective surface consists mainly of rigid ribs and reflective nets that can be folded radially. The tubular carbon-fiber ribs have 18 parabolic ribs uniformly fixed to a central hub, and the reflective net is fixed between these ribs. Typically, quartz tension lines are placed on the back of the reflective net to adjust the accuracy of the surface shape. Once in orbit, the antenna is controlled by a motor. As the rib cannot be folded, the height of the antenna is equal to the length of the rib. Although the radial rib deployable antenna has many advantages such as a simple structure, low weight, high reliability, and good reflector precision, the uneven stress distribution in the structure and the thermal deformation of the rib affect the whole antenna system.

According to the manufacturing process of the satellite antenna, the thermal deformation measurement test of the antenna reflector should be carried out in a high–low temperature environment to verify whether the design and manufacturing process of the satellite antenna meets the technical requirements of the product. For this reason, it is necessary to investigate the surface shape precision and deformation of the antenna in a high–low temperature environment. The antenna was placed in a high–low temperature simulation laboratory and counterweights were applied to simulate its operation in a high–low temperature space environment. The surface shape deformation of the antenna was then studied at different temperatures.

### 3.2. Measurement

The antenna with the retro-reflective target (RRT) was measured using the Chenway S36 digital camera. Close-range photogrammetry was carried out at the required temperature, and the three-dimensional spatial coordinates and surface shape accuracy of the antenna were obtained by data processing. The data results for the temperature node at +20 °C, in the middle of each cycle, were used as a reference for the analysis and comparison with other temperature nodes. The surface shape results obtained for each temperature node were then compared with the reference. The amount and law of surface shape deformation of each temperature node relative to the reference were then determined. This makes it possible to determine whether the requirements of the various technical indicators for antenna design and quality have been met.

Three measurement cycles were carried out in accordance with the technical specifications. The measurement process performed in each cycle was essentially the same. For example, the temperature range was −60 °C to +60 °C and the test temperature nodes were +20 °C, +60 °C, +20 °C, −20 °C, −60 °C, and −20 °C. These six temperature nodes represent a measurement cycle. Therefore, the thermal deformation measurement of the antenna surface shape was performed taking into account the operating conditions associated with these six temperature nodes. At the start of the experiment, the temperature in the test chamber was +20 °C. The temperature was then raised, cooled down three times, and then raised again, while maintaining a temperature difference of 40 °C between the nodes. When the temperature rises or falls to a particular temperature node, the temperature must be maintained for at least 2 h before photogrammetry of that temperature node can be performed.

In order to keep the surface shape of the satellite antenna stable at each temperature node, it was necessary to maintain the temperature for at least 2 h after increasing or decreasing the temperature to a particular temperature node before photogrammetry could be performed for that temperature node. A total of three measurement cycles were carried out.

1. Digital camera and its protection

A Chenway S36 digital camera (self-calibration via bundle adjustment) was used for the extreme temperature test. The other relevant parameters during acquisition were set to an aperture of F = 16 and an exposure time of T = 1/250 s. Camera-protection measures for close-range photogrammetry are described in reference [27].

2. Auxiliary light source

For the extreme temperature test, the SUNPAK AUTO DX 16R ring micro-flash was used as an auxiliary light source for photography [28]. Taking into account the influence of the shooting distance and environmental factors, the output flash intensity of this experiment was 1/128.

3. Retro-reflective target (RRT)

**Figure 4:**
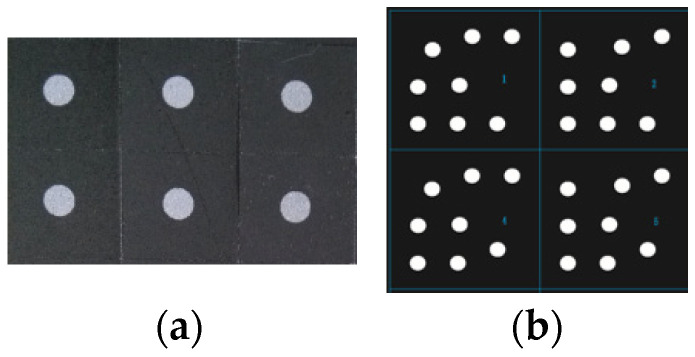
RRTs. (**a**) Single-point targets; (**b**) coded targets.

**Figure 5:**
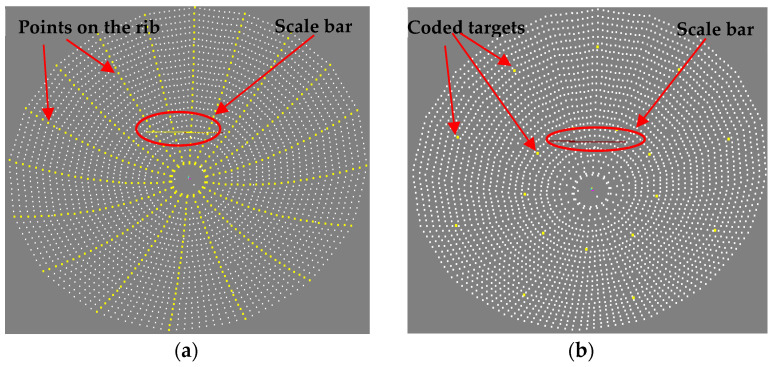
Distribution map of point locations on the reflective surface of the antenna. (**a**) Target points on the ribs (yellow dots); (**b**) coded targets (yellow dots).

**Figure 6:**
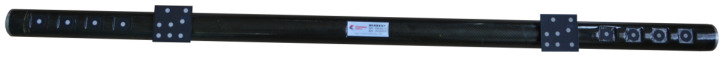
Carbon-fiber scale bar.

For close-range photogrammetry, two types of RRTs were attached to the reflective surface of the tested antenna. Specifically, a common single-point circular target and a coded target with unique coding information (Figure 4), as well as a total of 3073 RRTs with a diameter of 3 mm, were uniformly applied. A total of 18 coded targets were evenly distributed around the inner and outer circles of the antenna reflector (see yellow dots in Figure 5b). They are mainly used for fast image splicing and data processing automation. The remaining 3055 single-point RRTs were evenly distributed across the reflective surface of the antenna, as shown in Figure 6 with white and yellow dots.

The yellow single points in Figure 5a are the points positioned on 18 ribs of the reflective surface of the antenna. They were formed into a radial strip, and 27 single points were evenly spaced on each rib, making it easier to analyze the deformation of each rib as a function of temperature.

4. Scale bar

Accurate close-range photogrammetry requires a very stable and accurately calibrated reference length (see reference [29] for calibration methods) to provide a length reference for adjustments. A scale bar with a defined length is typically made of indium tile or carbon fiber. Given the extreme test environment, the coefficient of thermal expansion (mean: −4.3 × 10^−8^ m/°C) of the carbon-fiber scale bar (No. 025) was calibrated prior to the test. The test results show that the stability of the scale bar can meet the requirements of measurement accuracy. Therefore, the carbon-fiber scale bar, with a length of about 1 m, was selected as the length reference for close-range photogrammetry—see Figure 6. During the measurement it was fixed to the bracket above the antenna feed (Figure 5).

5. Photography and mesh design

**Figure 7:**
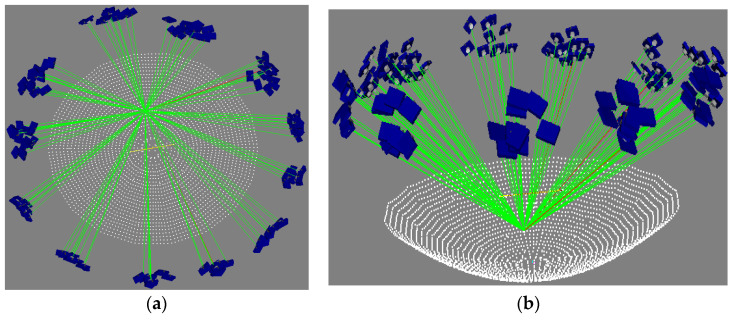
Mesh of antenna measurement. (**a**) Top view; (**b**) side view.

In this experiment, the best mesh geometry structure was used for photography according to the mesh optimization design strategy. The photographs were taken at a distance of approximately 2 m above the reflective surface of the antenna, and a total of approximately 125 images were taken at approximately 12 different locations. The camera was rotated four times at each location, by approximately 0°, 90°, 180° and 270°, to improve the geometry of the photographic space. The close-range photogrammetric mesh is shown in Figure 7.

6. Close-range photogrammetry data processing

A solution model was developed using collinear conditional equation imaging theory, and each photograph was digitally processed. This meant that the RRTs were automatically identified, extracted and located with high accuracy. Then, the plane coordinates for each image point were obtained and the image points with the same name were matched. Finally, the self-calibration bundle adjustment algorithm was used to calculate the three-dimensional coordinates of all the targets to be measured.

7. Post-processing of measurement data

To evaluate the effect and quality of the final measurements, and to check the surface shape deformation of the antenna, the surface shape registration post-processing method of the measurement data was used for processing and analysis to obtain the technical parameters. These parameters include surface shape accuracy and the surface shape deformation of the satellite antenna at the different temperature nodes. This was followed by thorough evaluation and feedback on the quality of the measurements obtained. The test object was a ribbed unfolded mesh satellite antenna with a caliber of about 4 m. Its surface shape was not a standard paraboloid. However, using the CAD model for the surface shape and a few design point coordinates, the fast iterative closest point (FICP) algorithm based on design coordinate guidance was selected [30]. To obtain the surface shape error (deviation) between the measured data at different temperature nodes and their CAD model, a fast fitting registration of the measured surface shape data for different temperature nodes (with its CAD model) was performed. The surface shape accuracy was then calculated. The surface shape error was used to derive the surface shape deformation of the other temperature nodes in each measurement cycle with respect to the reference temperature node (+20 °C).

## 4. Results and Analysis

The quality index for evaluating the measurement results of the close-range photogrammetry system was obtained by using the data processing and analysis. The quality indexes mainly include the coordinate repeatability measurement precision, the antenna surface shape repeatability measurement precision, and the length measurement error of the scale bar, which can reflect the stability and measurement accuracy of the close-range photogrammetry system. The quality evaluation indexes of the measured antenna mainly include the surface shape precision of the antenna reflective surface, the rib fitting precision, and the surface shape deformation. Considering the generality of the whole measurement process, this paper selects the measurement results of the three cycles of the middle second stage as an example to analyze; the first stage and the third stage have the same or similar measurement results with the second stage, and thus will not be analyzed excessively in the text.

### 4.1. Analysis of Close-Range Photogrammetry Results

#### 4.1.1. Coordinate Repeatability Measurement Precision

During the test, the antenna surface shape of each temperature node was measured three times in each measurement cycle of each stage. Each time, the coordinate repeatability measurement precision (RMS) was determined [28] for the three-dimensional coordinate results of the RRTs. The average of the RMS of the three measurement cycles at the same temperature was then obtained—see Table 1.

Table 1 shows that the three-dimensional coordinate repeatability measurement precision of the targets on the reflective surface of the antenna at different temperature nodes in the three cycles of this stage changes with temperature. Its coordinate repeatability measurement precision is best at 20 °C, with an average RMS of 0.027 mm, and worst at −60 °C, with an average RMS of 0.048 mm. Assuming the same accuracy for each temperature node, the single-point accuracy of close-range photogrammetry at the temperature nodes +20 °C, +60 °C, −20 °C, and −60 °C can be estimated to be approximately 0.019 mm, 0.023 mm, 0.023 mm, and 0.034 mm, respectively.

#### 4.1.2. Surface Shape Repeatability Measurement Precision

For repeated measurements at the same temperature node, the relatively short measurement time and the constant temperature in the test room (at least 2 h of heat preservation) mean that no new surface shape deformation of the measured antenna can be expected on a short time scale. The repeatability measurement precision (RMS) of the antenna surface shape can be calculated from the deviation of the surface shape between two repeated measurements. The results can express the precision of the measured surface shape (normal direction) for a close-range photogrammetry system—see Table 2.

Table 2 shows that the repeatability measurement precision of the antenna surface shape is relatively high, and the maximum RMS value does not exceed 0.04 mm. The results mainly reflect the measurement precision of the surface shape (normal direction), which is the one of practical significance.

#### 4.1.3. Scale Bar Length Measurement Error

The scale bar defines the length reference for a close-range photogrammetry system. The magnitude of the measurement error can directly reflect the accuracy of the measurement system and its effect on the final surface shape measurements. The carbon-fiber scale bar used in this experiment has four scales, each of which has been accurately calibrated by the metrology department. The calibration error of the carbon-fiber scale bar was about 3 μm. The calibrated length is considered to be the ‘true value’. During bundle adjustment, one of the scales was used as a length reference to calculate the measured lengths of the other three scales. The difference between the measured length and the ‘true value’ was also obtained. The difference between the lengths of the three scales was then determined. Finally, the average length difference of all the scale bars repeatedly measured at the temperature node was used as the measurement error for the scale bar length. The results are given in Table 3.

Table 3 shows that the change rule of the length measurement error of the scale bar with temperature is not obvious. The averages of the maximum and minimum errors are 0.039 mm and 0.014 mm, respectively. The results are consistent with the results of repeated measurements on the previous antenna surface shape and express the measurement accuracy of the close-range photogrammetry system.

### 4.2. Analysis of the Results for the Antenna Surface Shape

#### 4.2.1. Surface Shape and Rib Precision

**Figure 8:**
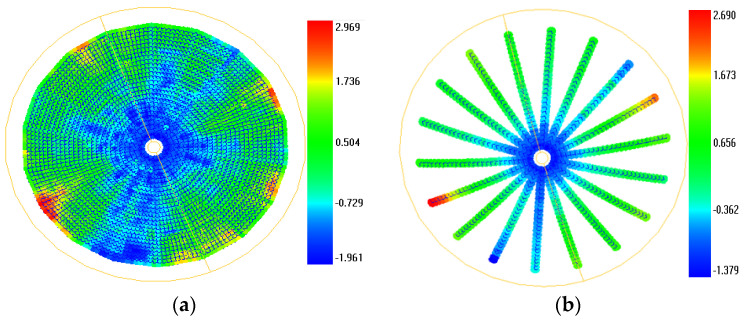
Deviation distribution for registration (units: mm). (**a**) Deviation distribution for the surface shape registration; (**b**) deviation distribution for the rib registration.

Since the intended surface shape of a satellite antenna is not a standard paraboloid, based on the known design information, the FICP free-form fitting registration method based on design coordinate guidance [30] was used to match the measured surface for different temperature nodes using its CAD model. The surface shape, rib, and Z-direction deviations were then determined for each target relative to its CAD model at different temperature nodes. The RMS of the surface shape, rib, and Z-direction deviations were then calculated. The resulting surface shape registration deviations for the temperature node −60 °C with the worst surface shape and rib precision in the first cycle of this stage are shown in Figure 8. The specific results of the mean precision of surface shape, rib, and Z deviation for all temperature nodes are shown in Table 4.

Figure 8 shows that in the lower left, upper right, center, and partial edge areas of the antenna, such as the red and blue areas, the registration deviation of the surface shape and rib of the antenna is large, while the surface shape and rib precision is poor. The blue and red areas indicate the convex and concave deformation of the designed antenna surface, respectively.

As can be seen from the results in Table 4, the precision of the antenna surface shape and ribs as well as the Z-direction deviation varies with the temperature change, and the change rule of the three cycles is more consistent. The surface shape and rib precision of the antenna deteriorate with decreasing temperature, and the surface shape and rib precision are worst at a temperature of −60 °C, with maximum RMSs of 0.878 mm and 0.761 mm, respectively. Moreover, the surface shape precision for the three cycles at the same temperature node deteriorates progressively. This suggests that the reversion of the antenna surface shape after continuous extreme thermal deformation is not sufficient. In addition, considering the previous test results of the close-range photogrammetric accuracy (less than 0.04 mm), and because the surface shape error mainly includes the photogrammetric error and the antenna surface shape deformation error, this shows that the deformation error for the antenna surface shape due to large temperature fluctuation is relatively high. Nevertheless, the requirements for a technical design index of less than or equal to 1 mm for the antenna surface shape accuracy are met.

#### 4.2.2. Surface Shape Deformation

**Figure 9:**
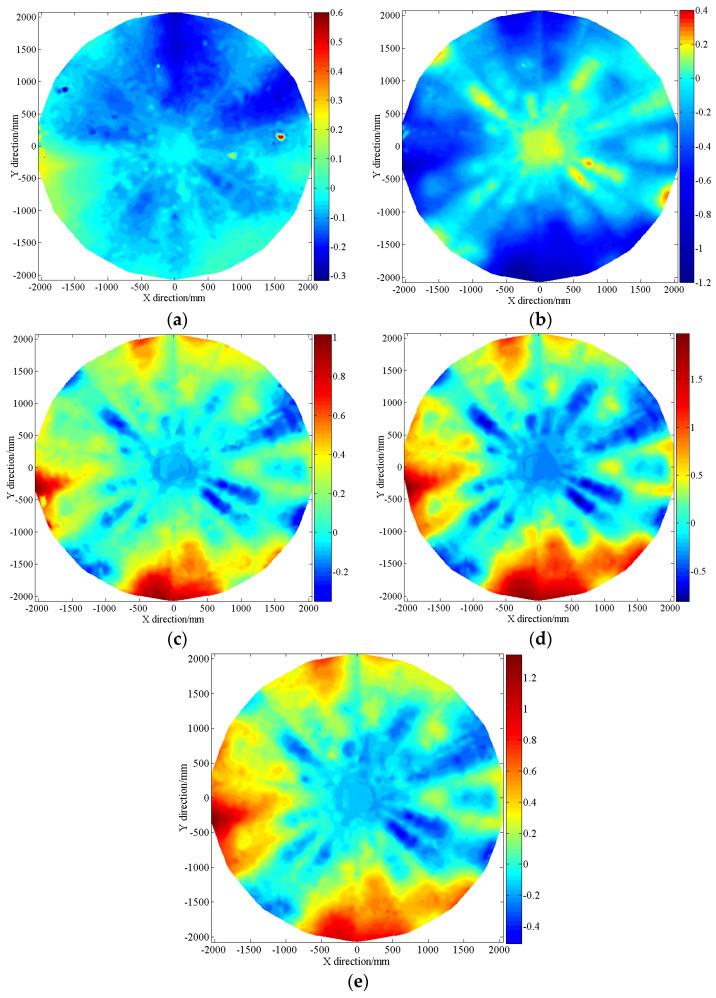
Deviation distribution for the surface shape and rib registration relative to reference +20 °C (R) (units: mm). (**a**) +20 °C VS. R.; (**b**) +60 °C VS. R.; (**c**) −20 °C VS. R.; (**d**) −60 °C VS. R.; (**e**) −20 °C VS. R.

From the surface shape error, it is possible to obtain the surface shape deformation of the other temperature nodes in each measurement cycle with respect to the intermediate temperature node (+20 °C) as a reference. The results of the surface shape deformation for the second cycle are shown in Figure 9 as an example limited by space. Note that data for a deformation greater than three times the RMS were considered outliers and deleted. The results for the total surface shape deformation of the three cycles are given in Table 5.

Figure 9 shows that when the temperature node (+20 °C) in the middle of each cycle is used as a reference, the positional distribution of the surface shape deformation region is consistent for each temperature node. In addition, the degree of deformation varies with the temperature change. In general, the deformation of the upper, lower, and left sides of the antenna is relatively large. Therefore, at high temperatures (Figure 9b), the surface shape of the antenna underwent severe sagging deformation due to thermal expansion. However, at low temperatures (Figure 9d), the surface shape of the antenna underwent a tensile upward deformation due to cold contraction.

The results in Table 5 show that the surface shape deformation laws for the three measurement cycles are consistent for this stage. In addition, the RMS of the surface shape deformation for each cycle is in the same order of magnitude for the same operating temperature node. The average RMS values of the surface shape deformation are 0.216 mm and 0.411 mm for the temperature differences of 40 °C and 80 °C, respectively, with respect to the temperature reference. These meet the technical design index requirements of 0.3 mm and 0.5 mm for the antenna surface shape deformation, respectively.

## 5. Conclusions

Based on the theoretical foundation of high-accuracy close-range photogrammetry, this paper successfully applied close-range photogrammetry technology to a thermal deformation measurement experiment of a satellite antenna surface shape. The whole process was carried out and described, including the design of the measurement scheme, the conduction of the experiment, and an analysis of the test results. Quantitative analysis and evaluation were carried out on the experimental results of close-range photogrammetry, and the ideal measurement accuracy was achieved, which meets the requirements of the measurement design technical index. At the same time, the quality and accuracy of the measurement results of the thermal deformation of the antenna surface shape were verified, which can meet the technical requirements of the thermal deformation measurement accuracy of the satellite antenna surface shape. This provides empirical guidance for further research on thermal deformation measurement tests of satellite antenna surface shapes, which has great practical significance and practical value.

## Figures and Tables

**Table 1:** Coordinate repeatability measurement precision.

Operating Condition (°C)	20	60	20	−20	−60	−20
RMS of three cycles (mm)	0.023	0.030	0.021	0.031	0.054	0.038
	0.033	0.034	0.036	0.036	0.040	0.029
	0.026	0.035	0.026	0.028	0.050	0.028
Mean value of RMS (mm)	0.027	0.033	0.028	0.032	0.048	0.032

**Table 2:** The repeatability measurement precision of the antenna surface shape.

Temperature Node (°C)	20	60	20	−20	−60	−20	20
RMS (mm)	0.019	0.014	0.016	0.021	0.038	0.015	0.019

**Table 3:** Measurement error of scale bar length.

Operating Condition (°C)	20	60	20	−20	−60	−20
Error results of three cycles (mm)	0.023	0.030	0.021	0.031	0.054	0.038
	0.033	0.034	0.036	0.036	0.040	0.029
	0.026	0.035	0.026	0.028	0.050	0.028

**Table 4:** Specific results of the mean precision of surface shape, rib, and Z deviation.

Cycles	Operating Condition (°C)	Surface Shape RMS (mm)	Rib RMS (mm)	Z Deviation RMS (mm)
1	20	0.476	0.379	−0.120
	60	0.352	0.348	−0.272
	20	0.512	0.420	−0.251
	−20	0.653	0.568	−0.473
	−60	0.817	0.707	−0.542
	−20	0.677	0.574	−0.412
2	20	0.562	0.442	−0.244
	60	0.455	0.331	−0.116
	20	0.518	0.415	−0.315
	−20	0.655	0.554	−0.490
	−60	0.866	0.761	−0.586
	−20	0.711	0.618	−0.492
3	20	0.562	0.452	−0.274
	60	0.472	0.343	−0.162
	20	0.533	0.423	−0.329
	−20	0.687	0.566	−0.572
	−60	0.878	0.742	−0.662
	−20	0.717	0.580	−0.498
	20	0.556	0.451	−0.294

**Table 5:** The mean RMS of the surface shape deformation.

Operating Condition (°C)	20	60	20	−20	−60	−20
RMS of three cycles (mm)	0.068	0.184	reference	0.202	0.419	0.246
	0.070	0.206		0.185	0.415	0.249
	0.058	0.230		0.194	0.399	0.251

## Data Availability

The data will be made available upon request.
